# Cerebro-Spinal Meningitis

**Published:** 1864-07

**Authors:** M. M. Eaton

**Affiliations:** Peoria, Ills.


### CEREBRO-SPINAL MENINGITIS.

By M. M. EATON. M. D., of Peoria, Ills.

It seems to be a fact that during the past year or 18 months, various parts of Illinois have been visited by an epidemic heretofore unknown experimentally in the West. The disease (to the best of my information,) first appeared near Galesburg, about 18 months since, and so fatal was it, that for a time the patients were supposed to have been poisoned.

In this vicinity it appeared about six months since, and now is upon the increase, though not so fatal as at first. I have interested myself very much in the disease, and have seen and corresponded with physicians upon it, from nearly all parts of the State. The symptoms vary somewhat, as in all other diseases, in different cases. My own experience is confined to 13 cases, several of which I have taken some of our oldest physicians to see. They have generally been attacked suddenly, without premonition, with a chill or a shivering; the countenance is shrunken and cadaverous; emesis soon sets in; delirium of a frightful type immediately follows; the pulse is rapid and weak; the pupil is for the most part dilated, with alternate contractions, and is frequently insensible to light; the bowels soon becomes tympanitic; the head is drawn back and great pain is complained of in some part or member, generally in one side of the head. Every movement of the body seems to give pain; the bowels are constipated; urine scanty and strangury is sometimes present; the temperature of the body is below the natural standard; the tongue bloodless; purple spots come and go on various parts of the body, in the outset in some cases, in others not till near death.

My cases have all been children from three to twelve years of age, save one case, a lady with two children. Other medical men in this city, however, have had adult males who were attaked with these symptoms and died. As to the causes I am ignorant. It has seemed to be infectious in some cases, attacking those who were much together. I have made but one autopsy. In this case the eyes were sunken; the membranes of the brain were intensely congested; there was effusion of lymph in the arachnoid; the mucous membrane of the stomach was softened and disorganized; other organs healthy.

I am informed by physicians from some dozen localities over the State, that their cases have been almost uniformly fatal when presenting the symptoms above enumerated. My own practice has been more favored, and for this cause I for a time felt uncertain that my cases were really of the same character as those who were proving so fatal with other practitioners, but am now certain that my cases were genuine attacks, and of the severest type. The first case I had died in 48 hours, without my being able to aid his recovery in the least. I then began my opium treatment, and since then have lost but three out of twelve cases, and to each of those that died I was called from 24 to 30 hours after delirium had set in, when I have no idea that anything can save them, when they have a genuine attack and have had no rational treatment before.

The treatment I have adopted (and I may say has been concurred in and adopted by the Medical Society of this city now, after having battled me some at first for using it,) is this, viz., to give opium as the sheet anchor in the first instance, as soon as called, if delirium has set in, and the pupil is dilated. Repeat the opium in doses according to the age of the patient, giving an adult 5 grs. to a dose every half hour or hour, until the patient falls into a good sound sleep, which in my adult case took about 40 grs. But no matter how much, look to the effect and not at the dose; allow the patient to sleep four or six hours, then give opii pulv., gr. ij; hydg. chlo. mit., gr. ss, every four hours, and immediately give tonics, tr. ferri chlo., small doses of sulph. quinia, brandy, beef tea, etc.

As soon as sleep is produced emesis ceases, and when the patient awakes he is generally rational, if not, continue the opium till he has another good sleep. He will then (i.e., after becoming rational,) be so weak he can hardly turn in bed; now give the tonics; keep the room still, slight noises annoy the patient exceedingly, and sometimes produce a relapse.

Dr. Murphy has used venesection in connection with the opium, in one case with good success, which, by the way, I consider good practice in some adult cases. I have used blisters to the back of the neck, and both anodyne and stimulating washes and liniments to the spine, without any perceptible benefit. The disease, methinks, is of course congestive in the beginning, affecting the membranes of the brain and spine, and primarily the arachnoid. Dr. Condie's article, in "Watson's Practice," on cerebro-spinal meningitis, is the best modern description of the disease that I have seen. In Copland's Dictionary of Prac. Med. I find a tolerably correct description of the disease, though less violent in the symptoms as a rule, than has been the epidemic from which we have been suffering. I learn from this work that the disease was first known in America in Medway, Mass., in 1806, and prevailed in the Eastern and Middle States, from that time to 1815. It prevailed all over Europe in 1505; also in 1528 and 1574; each of which times it was followed by the Plague. It occurred in Egypt in 1760, and in Switzerland in 1805. It has been every time confounded with other diseases, and probably many cures reported where the disease did not exist at all. I feel sad that we know so little of the disease as we do, as I believe every physician should be prepared to know it when he sees it, and strike hard blows in the outset, for it seems that after 24 hours the system is so prostrate that it is impossible to rally it again to normal action.

